# High-Sensitivity Plasmonic Temperature Sensor Based on a MIM Waveguide-Coupled TDSC Resonator

**DOI:** 10.3390/mi17020198

**Published:** 2026-02-01

**Authors:** Yuanyuan Gao, Shubin Yan, Hui Cai, Zhenyang Xu, Chen Chen, Guang Liu, Taiquan Wu

**Affiliations:** 1 College of Mechanical and Electrical Engineering, China Jiliang University, Hangzhou 310018, China; p24010858002@cjlu.edu.cn (Y.G.); caihui@cjlu.edu.cn (H.C.); 2 School of Electrical Engineering, Zhejiang University of Water Resources and Electric Power, Hangzhou 310018, China; cc@zuwe.edu.cn (C.C.); lg@zuwe.edu.cn (G.L.); wutq@zuwe.edu.cn (T.W.); 3 Joint Laboratory of Intelligent Equipment and System for Water Conservancy and Hydropower Safety Monitoring of Zhejiang Province and Belarus, Hangzhou 310018, China; 4 School of Geomatics Science and Technology, Zhejiang University of Water Resources and Electric Power, Hangzhou 310018, China; xzy1896@foxmail.com

**Keywords:** MIM, surface plasmon polaritons, refractive index sensor, Fano resonance, TDSC structure

## Abstract

This paper presents a nanoscale sensor based on a metal–insulator–metal (MIM) waveguide coupled with a composite resonant cavity, where the ring resonator is embedded with triangular, semicircular, and rectangular structural elements. The transmission characteristics and sensing performance of the structure were systematically analyzed using the finite element method. The results indicate that the interference between the continuous mode in the waveguide and the discrete mode in the resonant cavity generates a distinct asymmetric Fano resonance. The optimized sensor achieves a sensitivity of 2960 nm/RIU and a figure of merit (FOM) of 59.79. Experimental verification confirms that the structure exhibits high responsiveness in temperature sensing, providing an effective solution for integrated photonic devices.

## 1. Introduction

Surface plasmon polaritons (SPPs) can be described as a type of traveling wave confined to the interface of metallic and dielectric media, which is generated when incident photons couple with the free electrons in the metal. These waves travel along the interface while exhibiting exponential decay of their field distribution normal to the interface [1,2]. By virtue of their remarkable property of breaking through the classical diffraction limit, SPPs can confine optical energy at the nanoscale, thereby enabling effective manipulation and control of photon transmission at subwavelength dimensions [3,4]. Given these beneficial characteristics, SPPs show considerable promise for deployment in silicon-based optoelectronics, integrated optics, and associated functional components [5,6].

Utilizing the distinctive characteristics of surface plasmon polaritons (SPPs), a variety of novel photonic devices have been developed with nanoscale integration capabilities, and numerous SPP waveguide configurations have been successively proposed. Examples include strip waveguides [7,8], semiconductor–insulator–semiconductor (SIS) waveguides [9], metal–insulator–metal (MIM) waveguides [3], and dielectric–metal–dielectric (DMD) waveguides [10]. Owing to a set of advantages including straightforward geometry, facile excitation, strong field confinement, and low propagation loss, MIM waveguides stand out as a preferred platform for highly integrated SPP photonic devices. In recent years, plasmonic sensors based on metal–insulator–metal (MIM) waveguides have attracted significant attention due to their strong localized field enhancement, ease of integration, and excellent mode confinement capabilities. Particularly with the introduction of the Fano resonance mechanism, their sensing sensitivity and figure of merit (FOM) have been remarkably improved, offering new avenues for high-precision refractive index sensing, biomolecular detection, and environmental monitoring [11,12].

Furthermore, several distinctive optical phenomena have been observed in SPP waveguide systems, including plasmon-induced transparency (PIT) [13,14], Fano resonance [15,16], and electromagnetically induced transparency (EIT) [17,18]. In particular, the spectral signature of Fano resonance is a sharply asymmetric peak with a distinctly narrow full width at half maximum (FWHM), and demonstrates high sensitivity to variations in both waveguide structural parameters and the surrounding dielectric environment. Fano resonance is a characteristic scattering phenomenon that exhibits a distinct asymmetric line shape in its spectrum. Alterations in the system’s structural parameters or ambient refractive index typically induce a prominent shift in the Fano resonance spectrum [19]. Consequently, within the context of subwavelength structures, the combination of the unique spectral properties of Fano resonance with the structural advantages of metal–insulator–metal (MIM) waveguides enables precise control and optimization of the performance of photonic devices. Optimization of the dimensional parameters of both the MIM waveguide and the resonant cavity enhances the Fano resonance characteristics, consequently boosting the sensor’s final performance metrics.

Conventional ring or rectangular resonant cavities, while widely used, offer limited magnetic field localization and modal flexibility. This work introduces a novel design paradigm based on functional decomposition, wherein a single cavity is divided into specialized sub-units: sharp-angled components for energy injection and local enhancement, curved boundaries for resonance control and storage, and directional elements for phase-matched coupling. The integration of triangular, semicircular, and rectangular shapes within a ring (forming the TDSC structure) enables synergistic tailoring of the Fano resonance for advanced sensing. Furthermore, the design of high-performance plasmonic sensors often involves striking a balance between sensitivity (S) and the figure of merit (FOM). Certain structures achieve remarkably high sensitivity via specific resonant mode coupling mechanisms; however, this typically comes at the cost of a notably broadened resonant linewidth, resulting in a relatively lower FOM that may limit resolution when detecting minute refractive index changes. Conversely, other designs can attain a very high FOM, indicative of an extremely sharp resonance, yet their absolute sensitivity often leaves room for improvement. Consequently, developing a novel architecture capable of simultaneously delivering both high sensitivity and a high FOM is of significant importance for advancing high-precision nanophotonic sensing applications. The TDSC composite cavity structure proposed in this work directly addresses this goal. Through a synergistic field-localization design with multiple geometric degrees of freedom, it aims to achieve a substantial enhancement in absolute sensitivity without compromising the resonance linewidth.

This study presents a plasmonic nanoscale sensor utilizing surface plasmon polaritons (SPPs). The design integrates a metal–insulator–metal (MIM) waveguide with a composite circular cavity that contains triangular, semicircular, and rectangular elements (TDSC). Compared to conventional configurations, this multi-element embedded design enables more effective localization and enhancement of the magnetic field, thereby generating sharper Fano resonance spectra and higher sensing sensitivity. The transmission properties of the developed structure were evaluated through coupled-mode theory (CMT) [20,21] and finite element method (FEM) [22] simulations. Computational results demonstrate that the observed Fano resonance emerges from the interference of broadband propagating modes with discrete TDSC cavity modes. Comprehensive analysis of geometric and refractive index effects revealed optimal sensor performance characterized by 2960 nm/RIU sensitivity and 59.79 FOM. Furthermore, experimental validation confirms that the sensor exhibits excellent response characteristics in detecting temperature variations, ultimately demonstrating a highly sensitive refractive index sensing scheme.

## 2. Structural Model and Analysis Methods

**Figure 1:**
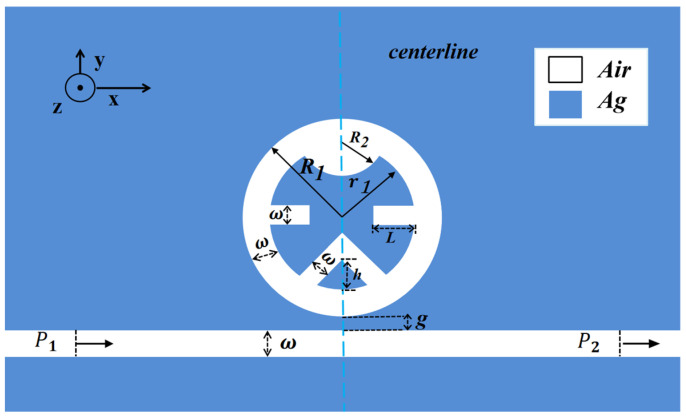
Two-dimensional schematic diagram of the MIM waveguide and the TDSC structure with a shielded cavity.

The schematic in Figure 1 depicts the designed sensor’s architecture. This integrated system is composed of a metal–insulator–metal (MIM) waveguide linked to a composite circular cavity incorporating triangular, semicircular, and rectangular elements (abbreviated as TDSC). Considering the substantial computational resources and refined mesh generation required for three-dimensional simulations, a two-dimensional approximate model is adopted in this study, as it effectively captures the essential magnetic field distribution characteristics. The 2D model neglects out-of-plane field variations and finite-thickness effects. This simplification is acceptable for the in-plane geometry optimization and comparative analysis central to this study. The entire structure exhibits vertical mirror symmetry, with the axis of symmetry passing through the center of the circular cavity. The 50 nm setting of the dielectric layer’s width *ω* in the MIM waveguide ensures exclusive excitation of the fundamental transverse magnetic (TM) mode. Definitions of other geometric parameters used in the model are summarized in Table 1.

In the diagram of Figure 1, white and blue colors indicate air and silver, respectively. Silver was chosen as the metal due to its optimal combination of stability, corrosion resistance, and low ohmic loss, facilitating efficient SPPs excitation in the MIM waveguide-coupled resonator system. The model defines air permittivity as 1 and characterizes silver dispersion via the Debye–Drude model, with the complete expression provided in Equation (1) [23].

(1) $$\varepsilon (\omega )=\varepsilon_{\infty }+\frac{\varepsilon_{s}-\varepsilon_{\infty }}{1+i\tau \omega }+\frac{\sigma }{i\omega \varepsilon_{0}}$$

In Equation (1), the key parameters for the silver’s permittivity model are given by: the infinite-frequency relative permittivity *ε*_∞_ = 3.8344; the relaxation time *τ* = 7.35 × 10^−15^ s; the conductivity *σ* = 1.1486 × 10^7^ S/m; and the static dielectric constant *ε*_s_ = −9530.5. The vacuum permittivity is denoted by *ε*_0_.

As illustrated in Figure 1, the structural design of the TDSC composite cavity is guided by distinct functional considerations. Specifically, the triangular element (height h) utilizes its sharp vertices to generate pronounced localized field enhancement, serving as a functional unit for efficiently exciting discrete modes. The semicircular element (radius R_2_), acting as the primary resonator, dictates the intrinsic resonant wavelength of the system through its curvature radius. The rectangular element (length L), by virtue of its straight edge being parallel to the bus waveguide, primarily governs the coupling efficiency between the continuous and discrete modes. Finally, the coupling distance g provides fine-tuning of the strength of energy exchange. This functionally decomposed design enables a systematic investigation into the individual contributions and synergistic effects of each geometric unit on the overall sensor performance.

The transverse magnetic (TM) mode excited during SPP propagation [24] can be described by the following Equation (2):

(2) $$tanh(k\omega )=-\frac{2k\alpha_{c}}{k^{2}+p^{2}\alpha_{c}}$$

where *k* represents the wave vector in the waveguide and is expressed in free space as *k*_0_ = 2π/λ_0_, *p* = *ε*_in_/*ε*_m_, *α*_c_ = [*k*_0_^2^(ε_in_ − ε_m_) + *k*^2^]^1/2^, ε_in_ and ε_m_ denote the permittivity values of the metal and dielectric, respectively.

Guided by standing wave theory, both the resonant wavelength and the real component of the effective refractive index within the MIM waveguide are determined using Equations (3) and (4) [25]:

(3) $$\lambda_{m}=\frac{2Re\left(n_{eff}\right)L}{\left(m-\frac{\psi_{r}}{\pi }\right)}\left(m=1,\;2,\;\ldots \right)$$

(4) $$Re(n_{eff})=\sqrt{\varepsilon_{m}+{\left(k/k_{0}\right)}^{2}}$$

In the formulation, *L* corresponds to the ring resonator’s perimeter, *ψ_r_* accounts for the phase shift produced by SPPs reflection at the metal-dielectric boundary, while m indicates the mode order as a positive integer.

Sensor performance is characterized by three critical criteria: full width at half maximum (FWHM), sensitivity (S), and figure of merit (FOM). FWHM is quantified as the resonance peak width at half-maximum amplitude, representing spectral narrowness. Sensitivity gauges the detection response to target changes, and FOM combines sensitivity with resolution capability. Their mathematical representations are provided as follows [26,27,28]:

(5) S=Δλ/Δn

(6) FOM=S/FWHM

In this formulation, Δ*λ* and Δ*n* signify the respective alterations in resonance wavelength and refractive index. The sensitivity S is defined with units of nm/RIU.

Given the expense and complexity of nanofabrication, numerical simulation plays a paramount role. It allows for the flexible design and thorough optimization of sensor architectures, thereby maximizing their potential performance before physical realization. For precise structural analysis of the sensor’s optical characteristics, a two-dimensional model was constructed through simulations in COMSOL Multiphysics 5.4a. In the simulation setup, the input port was excited by a highly monochromatic and coherent laser source, with energy injected into the system through this port, while the output port was set in an unexcited state to monitor the transmission spectrum. All boundaries besides the input/output ports were assigned as perfectly matched layers (PML), ensuring reliable simulations through effective absorption of scattered radiation and suppression of boundary reflections. Meshing operations employing ultra-fine triangular elements were implemented in the MIM waveguide and TDSC sections to boost calculation accuracy in these critical areas. The mesh dimensions were adaptively adjusted based on structural features, ranging from the nanometer to sub-nanometer scale, thereby effectively capturing localized field distributions and boundary effects at the nanoscale while maintaining computational efficiency. The results confirmed that further refinement (e.g., halving the maximum element size) led to a shift in the key resonance wavelength of far less than 1 nm, which is negligible within the scope of our performance analysis. The finite element method (FEM) was chosen for this study primarily because of its capability to handle the complex curved boundaries and multi-scale features of the TDSC structure, enabling precise local mesh control and accurate implementation of material boundary conditions. Spectral scanning parameters covered 1600–2800 nm with 1 nm increments, allowing thorough analysis of the sensor’s optical response throughout this band. This simulation configuration provides a reliable basis for evaluating sensor performance and facilitates the detection of potential optical resonance phenomena.

## 3. Simulation Results and Analysis

**Figure 2:**
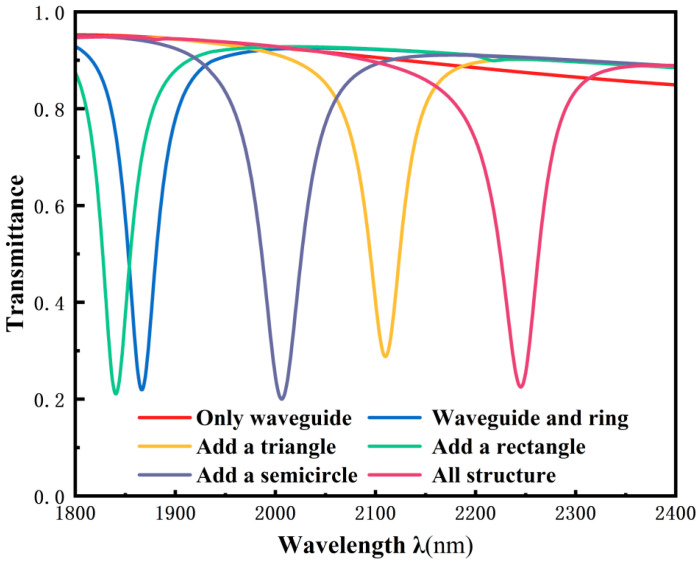
Transmission spectra of different structures.

In nanoscale sensing applications, Fano resonance serves as an indispensable component for characterizing wave propagation features and precisely evaluating key parameters including sensitivity and FOM. Accordingly, initial investigation focuses on Fano resonance’s origin and spectral manifestations. Figure 2 shows the respective simulation results comparing transmission spectra of the rectangular waveguide and TDSC structure. The colored traces depict transmission responses for: waveguide only (red), single ring (blue), ring-triangle (yellow), ring-rectangle (green), ring-semicircle (purple), and complete TDSC (magenta). It can be observed that the transmission spectrum of the complete TDSC structure exhibits a distinct asymmetric sharp peak, which is a typical characteristic of Fano resonance. This phenomenon originates from the interference between the continuous broadband mode and the discrete narrowband mode. The red curve (single rectangular waveguide) exhibits a monotonically increasing spectral profile with elevated, uniform transmittance, representing the fundamental broadband mode. Conversely, introducing triangular, semicircular, or rectangular elements generates localized narrowband resonances that form the discrete components of Fano resonance.

**Figure 3:**
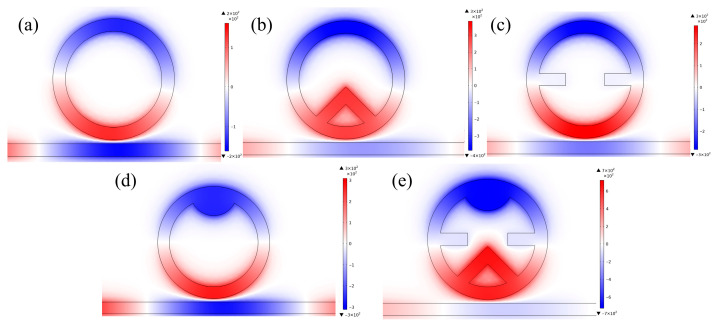
Magnetic field intensity distributions: (**a**) waveguide and ring; (**b**) with triangular structure; (**c**) with rectangular structure; (**d**) with semicircular structure; (**e**) TDSC structure.

To gain deeper insight into Fano resonance generation, we examine magnetic field patterns at spectral dips for five structural variants: the single-ring structure, ring–triangle, ring–rectangle, ring–semicircle, and the complete TDSC structure. Simulation results confirm efficient SPP transmission along the bus waveguide with effective coupling to all five configurations. In Figure 3a, despite generally low field intensity (especially in the bus region), normalized fields primarily localize in the ring cavity’s upper and lower sections, achieving 2140 nm/RIU sensitivity. In Figure 3b–d, the incorporated micro-nano configurations act as magnetic field localization sites, focusing additional SPP energy into the ring cavity thus improving device functionality. Subsequent analysis of the TDSC composite configuration (Figure 3e), the synergistic effect of multiple microstructures leads to significant magnetic field concentration along the symmetry direction and a notable enhancement in coupling strength. The magnetic field is particularly concentrated near the tip of the triangle, the inner boundary of the semicircle, and the edges of the rectangle. This distribution corresponds well to the intended functions: the triangular structure effectively enables energy injection and localization enhancement; the semicircular structure sustains the primary ring resonance mode; while the rectangular structure facilitates directional energy transfer from the waveguide to the cavity and concurrently suppresses retro-reflection.

The normalized magnetic field is predominantly confined within the TDSC composite cavity, with only minimal distribution in the bus waveguide (Figure 3e). This intense localization signifies a pronounced resonance effect, achieved through the synergistic confinement of all three structural elements, which enables superior energy concentration. Furthermore, it indicates highly efficient energy coupling from the waveguide into this hybrid resonant mode, accompanied by effective suppression of back-reflection. This synergistic mechanism directly corresponds to the sharper Fano resonance lineshape and the enhanced sensitivity observed in the transmission spectrum. Ultimately, this configuration achieved optimal sensitivity at 2960 nm/RIU, demonstrating outstanding sensing performance.

**Figure 4:**
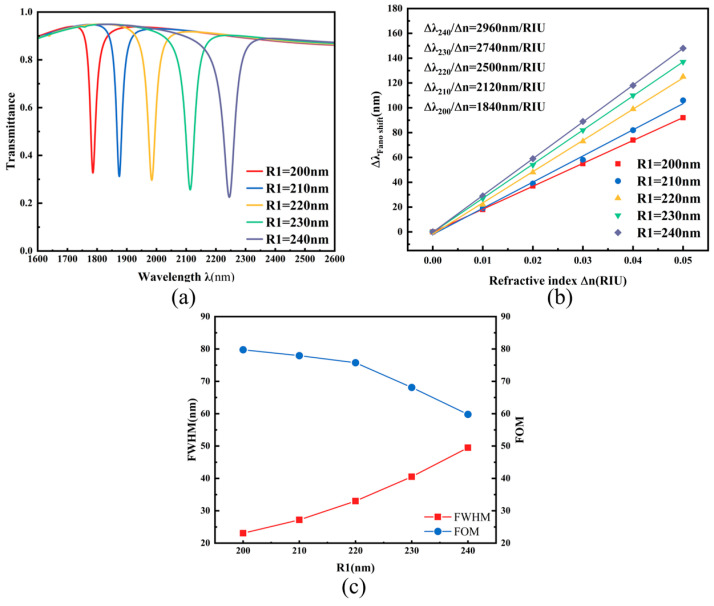
(**a**) Transmission spectra under different outer radii R_1_; (**b**) Sensitivity fitting lines for different outer radii R_1_; (**c**) Comparison of FWHM and FOM under different outer radii R_1_.

Fano resonance results from the interference between a continuum (bright) waveguide mode and a discrete (dark) cavity mode, with its lineshape being a function of their coupling strength and frequency detuning. This work employs systematic variation in geometric parameters to mimic the tuning of these underlying physical variables: the coupling gap *g* predominantly controls the coupling strength, and cavity sizes (e.g., R_1_) alter the resonant frequency of the discrete mode, thereby introducing detuning. How these geometric tuning knobs quantitatively affect the ultimate sensor performance is elucidated in the analysis below. Initial analysis addressed the TDSC’s outer radius R_1_: under fixed values of other parameters, R_1_ was incrementally adjusted from 200 nm to 240 nm in steps of 10 nm. With progressive enlargement of R_1_, Figure 4a reveals a noticeable redshift in the transmission spectrum, concurrent with reduced transmission intensity and progressive FWHM broadening. This red shift indicates an improvement in the overall structural sensitivity, while the slight increase in FWHM suggests an extended dwell time of the optical wave near the resonant frequency, which helps enhance the signal response and further improve the detection sensitivity. The parametric relationship between sensitivity and radius is quantified in Figure 4b via linear fitting. Sensitivity shows substantial improvement from 1840 nm/RIU to 2960 nm/RIU with increasing R_1_. Meanwhile, the blue curve in Figure 4c reflects the variation trend of another performance metric—the figure of merit (FOM)—which shows a gradual decline, opposite to the trend of sensitivity. This result indicates that a trade-off between sensitivity and FOM is necessary in practical sensor design. After comprehensive consideration, the resonator demonstrates optimal overall performance when R_1_ = 240 nm.

**Figure 5:**
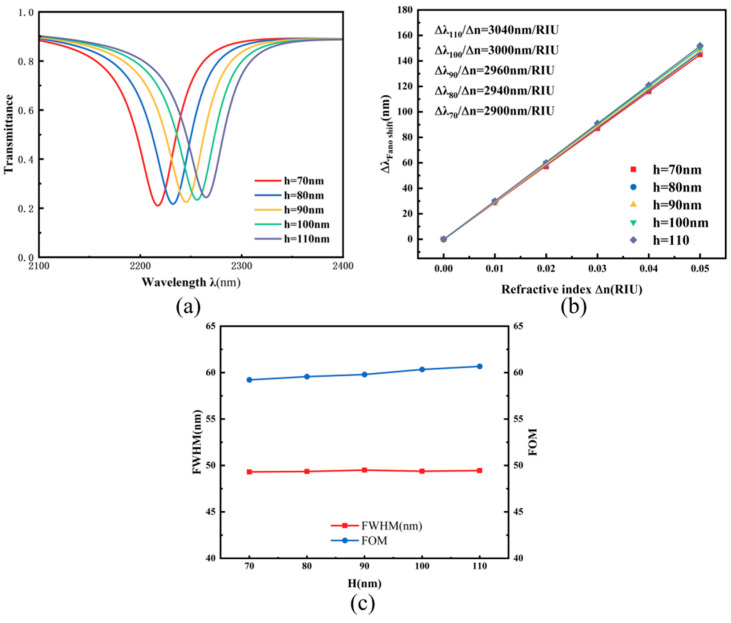
(**a**) Transmission spectra under different heights h; (**b**) Sensitivity fitting lines for different heights h; (**c**) Comparison of FWHM and FOM under different heights h.

Systematic evaluation of sensor response to auxiliary cavity dimensions commenced with analyzing the role of the triangular structure’s height h. Under conditions where all other parameters remained fixed, h was increased from 70 nm to 110 nm in 10 nm increments. As shown in Figure 5a, a slight redshift in the transmission spectrum is observed as h increases. According to the definition of sensitivity, this redshift indicates an improvement in the overall system sensitivity under identical refractive index variations. The sensitivity relationship with h parameter in Figure 5b, established via linear regression, indicates a steady gain from 2940 nm/RIU to 3020 nm/RIU accompanying the triangular structure’s height augmentation. Figure 5c illustrates the behavior of both resonance linewidth and quality factor across varying h values, showing near-constant values for each metric. Consequently, the triangular element’s height can be adapted to meet particular sensing needs.

**Figure 6:**
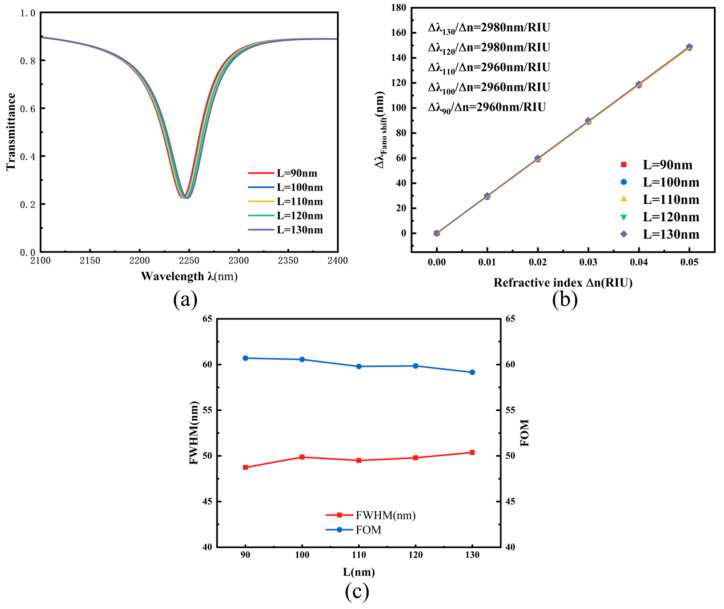
(**a**) Transmission spectra under different rectangular widths L; (**b**) Sensitivity fitting lines for different rectangular widths L; (**c**) Comparison of FWHM and FOM under different rectangular widths L.

Subsequent analysis addressed the role of dimension L in shaping optical transmission. With other dimensions fixed, the rectangular element’s width underwent stepwise enlargement from 90 nm to 130 nm using 10 nm steps. Figure 6a,b display the corresponding spectral transmission data and sensitivity fitting outcomes. Extension of the rectangular component induces a minor bathochromic shift in resonant features, though spectral lineshape and resonance linewidth maintain stability. Figure 6b indicates a modest improvement in system sensitivity with increasing rectangular width. Figure 6c further illustrates the variations in FWHM and figure of merit (FOM) with respect to the rectangular width, both of which remain essentially constant. Consequently, the rectangular dimensions can be appropriately adjusted according to structural requirements in practical applications without significantly affecting the sensor’s sensitivity.

**Figure 7:**
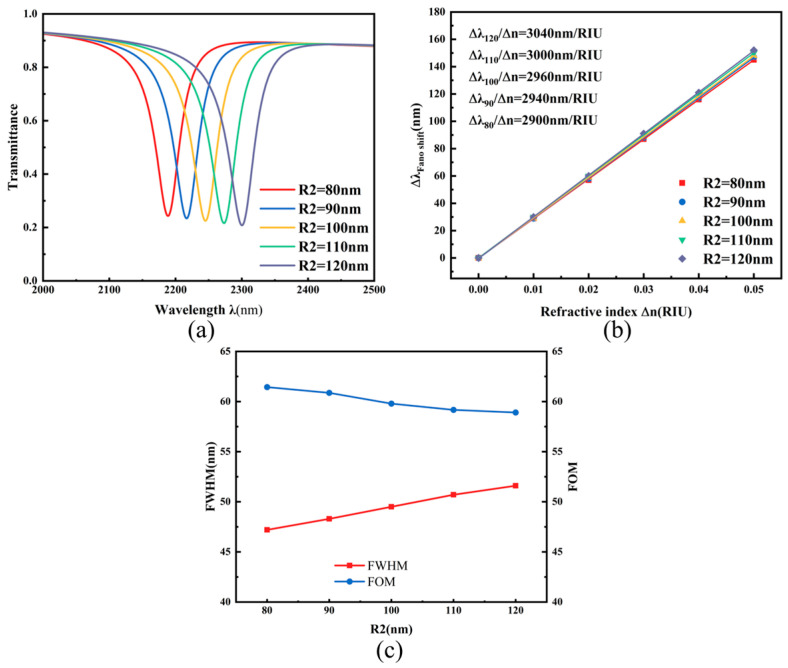
(**a**) Transmission spectra under different outer radii R_2_; (**b**) Sensitivity fitting lines for different outer radii R_2_; (**c**) Comparison of FWHM and FOM under different outer radii R_2_.

To further investigate the influence of the semicircular resonant cavity dimensions on sensor performance, we systematically analyzed the optical response as the radius R_2_ was varied from 80 nm to 120 nm in 10 nm increments. As shown in Figure 7a, the transmission spectrum exhibits a distinct redshift with increasing R_2_, accompanied by a slight decrease in transmittance, indicating that structural modifications of the semicircular cavity effectively modulate the resonant characteristics of the system. Figure 7b displays the sensitivity fitting results corresponding to different R_2_ values. As the radius increases from 80 nm to 120 nm, the sensitivity gradually improves from 2900 nm/RIU to 3040 nm/RIU, though the overall variation remains relatively moderate. Figure 7c further illustrates the variation trends of the full width at half maximum (FWHM) and figure of merit (FOM) with respect to R_2_, where FWHM and FOM exhibit opposing trends as R_2_ increases. Consequently, the dimension of R_2_ can be flexibly selected in practical device design according to specific transmittance requirements. Considering all performance metrics, the sensor achieves an optimal balance between sensitivity and FOM when R_2_ = 100 nm, with a corresponding sensitivity of 2960 nm/RIU and an FOM of 59.79.

**Figure 8:**
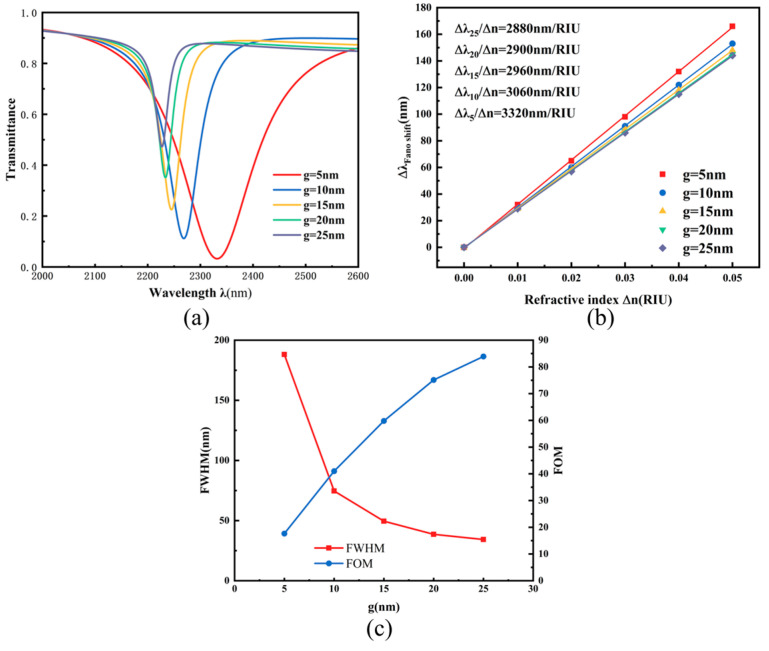
(**a**) Transmission spectra under different coupling distances g; (**b**) Sensitivity fitting lines for different coupling distances g; (**c**) Comparison of FWHM and FOM under different coupling distances g.

To gain deeper insight into the coupling mechanism between the TDSC structure and the main waveguide, the influence of the coupling gap g on the transmission characteristics was systematically investigated. This parameter directly determines the energy exchange efficiency between the two components. Under fixed values of other structural parameters, g was progressively increased from 5 nm to 25 nm for simulation analysis. The results reveal a clear regulatory effect of g on the optical response. As shown in Figure 8a, the transmission spectrum exhibits a significant blueshift along with a notable increase in transmittance as g increases. This phenomenon indicates that a larger coupling gap hinders the efficient coupling of surface plasmon polaritons (SPPs) into the resonant cavity, causing more energy to be directly transmitted through the output port and resulting in a weakened electric field within the cavity. Figure 8b shows that although the general trend of the sensitivity fitting curve remains consistent across different g values, the system sensitivity gradually decreases from 3320 nm/RIU to 2880 nm/RIU as g increases. Furthermore, Figure 8c indicates that when g < 10 nm, the full width at half maximum (FWHM, red curve) increases sharply, while the figure of merit (FOM, blue curve) exhibits an opposite trend. Comprehensive analysis demonstrates that the selection of the coupling distance g requires a careful trade-off between the FWHM and FOM. If g is too small, it leads to resonance peak broadening and a reduced FOM; if g is too large, the transmittance becomes excessively high, resulting in insufficient resonance depth. Taking all factors into account, an optimal balance of performance metrics is achieved when g = 15 nm.

The systematic parametric studies (Figure 4, Figure 5, Figure 6 and Figure 7) elucidate the distinct roles of each functional unit within the TDSC composite cavity: the outer radius R_1_ governs the effective range of the resonant mode; the triangular height h finely regulates the strength of localized field enhancement; the semicircular radius R_2_ enables precise tuning of the resonant wavelength; the rectangular length L directly influences the mode coupling efficiency and the internal field distribution; and the coupling distance g ultimately balances the coupling strength against radiative loss. Collectively, these findings demonstrate that the TDSC design offers multiple, independently tunable degrees of freedom for performance optimization—an advantage unattainable with conventional simple ring cavities or cavities modified with only a single element.

**Figure 9:**
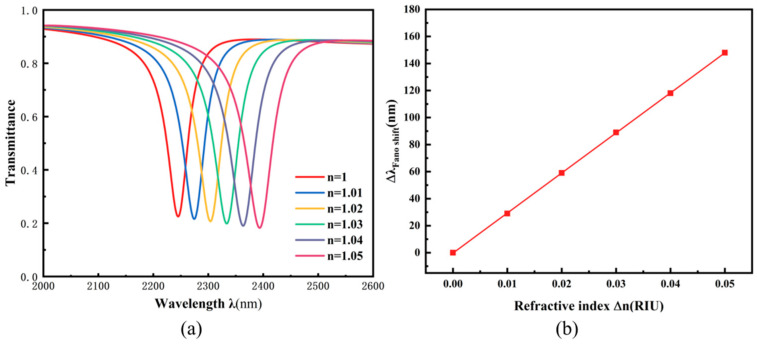
(**a**) Transmission spectra under different refractive indices n; (**b**) sensitivity fitting lines for different refractive indices n.

Based on systematic optimization studies, the optimal structural parameters of the sensor were determined as follows: g = 15 nm, R_1_ = 240 nm, R_2_ = 100 nm, h = 90 nm, and L = 110 nm. With these parameters established, modifying the environmental refractive index effectively tunes the wavelength position of the Fano resonance dip, demonstrating a well-defined linear dependence between the two variables. This relationship constitutes the fundamental operating principle enabling refractive index sensing with this structure. In practical sensing applications, where the refractive index of analytes typically varies within a certain range, it is essential to evaluate the sensor’s response performance across different refractive indices. The environmental refractive index was systematically increased from 1.00 to 1.05 in 0.01 increments to analyze its effect on sensor performance, with results presented in Figure 9. Figure 9a shows that as the refractive index increases, the transmission spectrum exhibits a nearly equidistant redshift while maintaining essentially identical line shapes, indicating that refractive index variations primarily cause resonance wavelength shifts without altering the fundamental resonance mode. Figure 9b further confirms a distinct linear relationship between the resonance dip wavelength and the refractive index. The sensitivity, calculated from the slope of the linear fit, reaches 2960 nm/RIU with a figure of merit (FOM) of 59.79. As summarized in Table 2, the performance of the optimized TDSC sensor is benchmarked against various recently reported plasmonic architectures. Notably, the TDSC structure strikes an excellent balance between a sensitivity of 2960 nm/RIU and a figure of merit (FOM) of 59.79. While some individual structures may achieve a higher value in a single specific metric, the TDSC design delivers considerable sensitivity while simultaneously maintaining the high FOM, which is indicative of a sharp resonance peak and high resolution.

## 4. Application

Plasmonic temperature sensors typically transduce thermal changes via the thermo-optic effect of a dielectric medium filling the sensor. Thermal sensing operates through the thermal dependence of refractive index in the sensitive medium, which modifies its optical properties with temperature changes and leads to corresponding spectral displacements. Reported sensitivities for such MIM-based sensors vary widely, ranging from below 0.1 nm/°C to over 3.0 nm/°C [34,35], depending on the structure and the thermo-optic coefficient of the filling medium. In this context, the optimized TDSC sensor, due to its high refractive index sensitivity, is well-suited for temperature detection. The sensor design shows high sensitivity and integration potential in simulation, and is favorable for standard nanofabrication such as forming the microstructure by magnetron sputtering and etching of a silver film on quartz. Ethanol, possessing a substantial thermo-optic coefficient (3.94 × 10^−4^ °C^−1^), serves as the detection medium filled into both the TDSC configuration and MIM waveguide. The device employs silver and quartz as the metal and substrate materials, possessing thermo-optic coefficients of 9.30 × 10^−6^ °C^−1^ and 8.60 × 10^−6^ °C^−1^. These values are substantially smaller than ethanol’s coefficient, making the liquid’s refractive index the primary factor affected by thermal fluctuations. Across ethanol’s liquid phase range (−144 °C to 78 °C), its refractive index varies linearly with temperature according to [36]:

(7) *n* = 1.36048 − 3.94 × 10^−4^ × (*T* − *T*_0_)

The model defines *T* as ambient temperature with *T*_0_ = 20 °C as the reference. *T*_0_ maintain operational stability within ethanol’s phase transition thresholds, the sensor’s working range is confined between −80 °C to 70 °C. Although ethanol has a melting point as low as −144 °C, and the sensor is theoretically capable of detecting temperatures down to approximately −130 °C, measurement accuracy degrades and errors increase significantly when the temperature falls below −80 °C or exceeds 70 °C. The optimized structure with a sensitivity of 2960 nm/RIU was selected for temperature testing. The temperature was progressively lowered from 70 °C to −80 °C in 30 °C steps. Ethanol’s refractive index variation was determined by monitoring the spectral displacement, thus facilitating temperature detection. The thermal sensitivity *S*_T_ is obtained from:

(8) *S*_T_ = ∆*λ*/∆*T*

**Figure 10:**
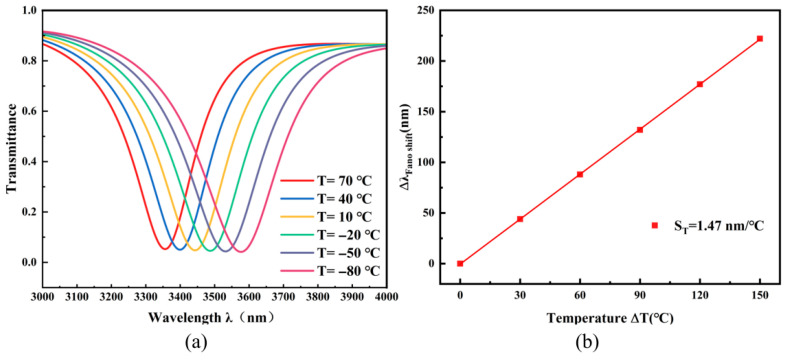
(**a**) Transmission spectra measured across the temperature range from −80 °C to 70 °C; (**b**) Sensitivity fitting curve of the temperature sensor.

Over the investigated thermal interval, the medium’s refractive index spans 1.34078 to 1.39988. Corresponding spectral responses at distinct temperatures are presented in Figure 10a. The resonance wavelength exhibits a systematic redshift with decreasing temperature, confirming the structure’s high thermo-optic sensitivity. Experimental measurements record a resonance dip displacement from 3355 nm to 3577 nm, yielding a total spectral shift (Δ*λ*) of 222 nm. As shown in Figure 10b, the relationship between temperature and wavelength shift exhibits excellent linearity, ensuring measurement reliability. The calculated temperature sensitivity *S*_T_ is 1.47 nm/°C. The structural design exhibits excellent temperature sensing capability due to its high sensitivity, though current manufacturing constraints for nanoscale features create a mismatch between modeled and actual performance. This disparity is projected to decrease with ongoing improvements in nanofabrication methodologies. The demonstrated high sensitivity makes the TDSC structure a promising candidate for integrated photonic sensors. Future work will focus on the practical implementation, which includes addressing engineering aspects such as integration with microfluidic systems for stable liquid handling and packaging for robust operation.

## 5. Conclusions

We propose a plasmonic nanoscale sensing device employing surface plasmon polaritons. The design incorporates a metal–insulator–metal (MIM) waveguide coupled with a composite circular resonant cavity (TDSC). The waveguiding properties and sensing principles of the proposed configuration were analyzed through finite element simulations and coupled-mode theory. Analysis reveals that Fano resonance results from the interaction between the waveguide’s broadband continuum and the cavity’s localized resonances, with its spectral characteristics controlled via critical dimensions including outer radius R_1_ and coupling gap g. Following parametric optimization, the sensor achieves 2960 nm/RIU and a FOM of 59.79 in refractive index sensing. It also displays excellent linear response in temperature detection, reaching 1.47 nm/°C. This study establishes a viable approach to designing highly sensitive nanophotonic sensors and indicates considerable promise for integrated photonic systems.

## Figures and Tables

**Table 1:** Parameter Definitions for the Structure.

Parameter	Definition	Optimal Value (nm)
R_1_	Outer radius of the ring	240
r_1_	Inner radius of the ring	190
R_2_	Radius of the semicircular disk	100
h	Height of the triangle	90
L	Length of the rectangle	110
g	Coupling distance	15
ω	Waveguide width	50
P_1_	Optical input port	/
P_2_	Optical output port	/

**Table 2:** Performance comparison with previously reported sensor architectures.

Structure Type	Sensitivity (nm/RIU)	Figure of Merit (FOM)	Wavelength Range (nm)
SERR [29]	1783	27	500 < λ < 2000
Nano ring resonator [30]	2080	29.9	1000 < λ < 2600
Bow-tie resonator [31]	2300	31	800 < λ < 2300
SRS [32]	6000	17	600 < λ < 2800
BW/CSRR [33]	1200	53	600 < λ < 1800
This work	2960	59.79	1600 < λ < 2800

## Data Availability

The data used to support the findings of this study are available from the corresponding author upon reasonable request.
